# Fundus images analysis using deep features for detection of exudates, hemorrhages and microaneurysms

**DOI:** 10.1186/s12886-018-0954-4

**Published:** 2018-11-06

**Authors:** Parham Khojasteh, Behzad Aliahmad, Dinesh K. Kumar

**Affiliations:** Biosignal Lab, School of Engineering, RMIT University, Melbourne, Australia

**Keywords:** Fundus image analysis, Diabetic retinopathy, Deep learning, Convolutional neural networks, Image processing

## Abstract

**Background:**

Convolution neural networks have been considered for automatic analysis of fundus images to detect signs of diabetic retinopathy but suffer from low sensitivity.

**Methods:**

This study has proposed an alternate method using probabilistic output from Convolution neural network to automatically and simultaneously detect exudates, hemorrhages and microaneurysms. The method was evaluated using two approaches: patch and image-based analysis of the fundus images on two public databases: DIARETDB1 and e-Ophtha. The novelty of the proposed method is that the images were analyzed using probability maps generated by score values of the softmax layer instead of the use of the binary output.

**Results:**

The sensitivity of the proposed approach was 0.96, 0.84 and 0.85 for detection of exudates, hemorrhages and microaneurysms, respectively when considering patch-based analysis. The results show overall accuracy for DIARETDB1 was 97.3% and 86.6% for e-Ophtha. The error rate for image-based analysis was also significantly reduced when compared with other works.

**Conclusion:**

The proposed method provides the framework for convolution neural network-based analysis of fundus images to identify exudates, hemorrhages, and microaneurysms. It obtained accuracy and sensitivity which were significantly better than the reported studies and makes it suitable for automatic diabetic retinopathy signs detection.

## Background

**Fig. 1:**
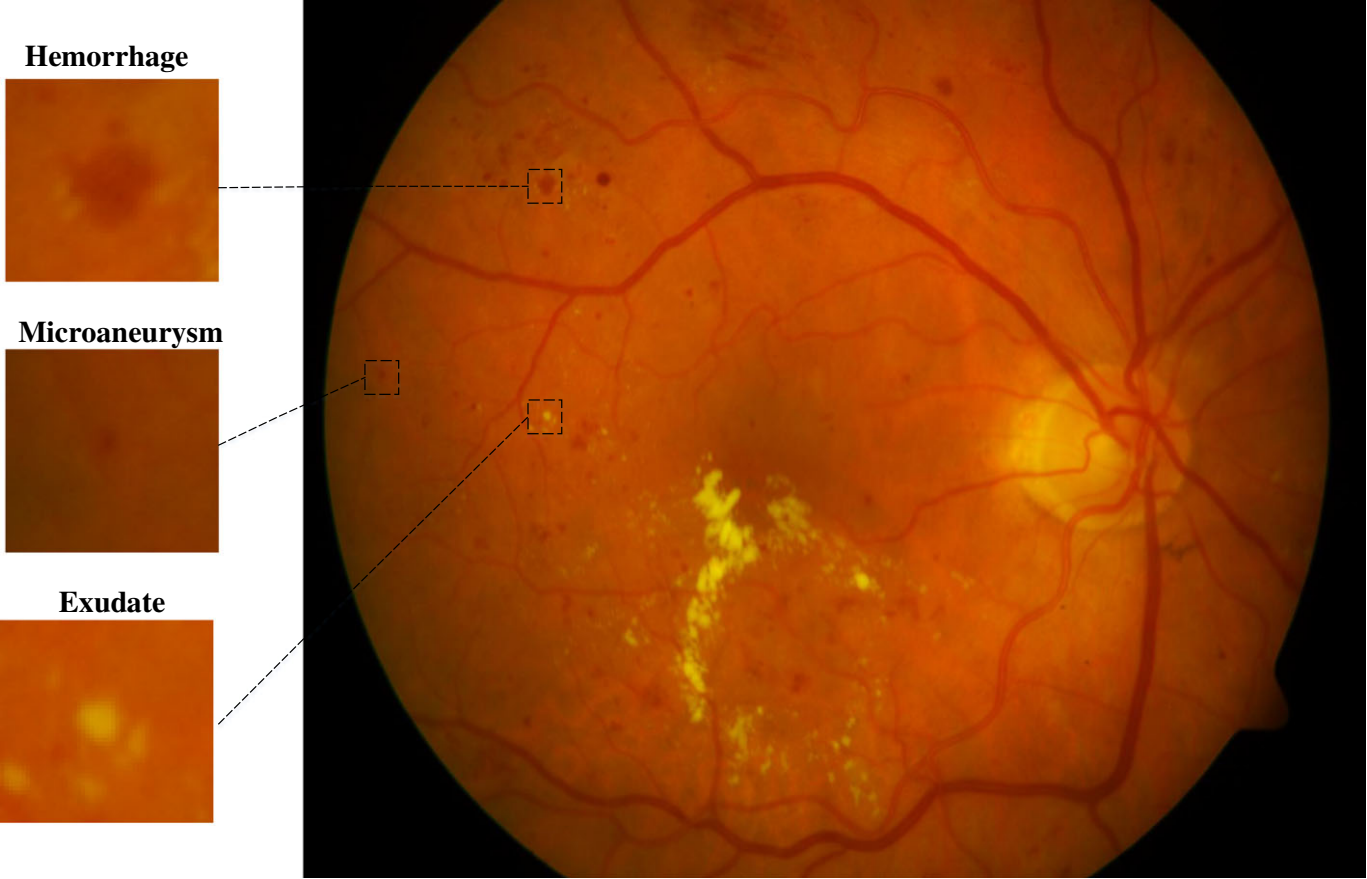
Example of Retina Images containing three DR sings. This image shows an entire retina image with haemorrhage, microaneurysm and exudate labled by graders, and which was then cropped to illustrate individual patch

Diabetic retinopathy (DR) is a leading cause of vision impairment and irreversible blindness in middle-aged and elderly people [1, 2] and is expected to rise to 191 million by 2030 [3–5]. Vision impairment due to DR can be significantly reduced if it is diagnosed in the early stages. It is diagnosed by visual examination of retinal images to detect three most common pathological signs i.e. (i) exudate (ii) hemorrhage and (iii) microaneurysm [6]. However, this is a manual time-consuming procedure and outcomes are subjective and dependent on expertise, thus, there is potential bias of the examiner. The diagnosis can be performed by analysis of color fundus images or fluorescein angiograms (FA) to identify pathological signs. Although FA enables better differentiation between microaneurisms and micro hemorrhages, due to its invasive nature along with costs and the risk of allergic reactions, fundus images are the preferred modality. For automatic detection of pathological signs, most computer-based studies have developed algorithms for the automatic analysis of the fundus images with the aim to make the diagnosis more objective and easier to access by people in remote communities. However, this is very challenging because of variation in size, color, texture and shape of these signs (Fig. 1).

In computer-based methods, detection of exudate, hemorrhage and microaneurysm can either be done separately for each signs [7–23] or all signs simultaneously [24–31]. For exudate detection, Sánchez et al. [32] used a statistical mixture model-based clustering for dynamic thresholding to separate exudate from background. The algorithm obtained sensitivity of 90.2% and 96.8% for lesion and background, respectively. Giancardo et al. [7] proposed a method based on color and wavelet decomposition features from exudate candidates to train classifiers. They achieved the best result using support vector machine (SVM) classifier with areas under the receiver operating characteristics (AUC) between 0.88 and 0.94, depending on different datasets. In 2017, Fraz et al. [8] developed a method to detect exudate based on the multiscale segmentation. They used combination of morphological reconstructions and Gabor filter banks for feature extraction followed by bootstrap decision tree for classification of exudate pixels. In 2018, Kaur and Mittal [3] used a dynamic thresholding method for detection of exudate boundaries. The algorithm obtained sensitivity of 88.85% and 94.62% in lesion-based and image-based, respectively.

Hemorrhage detection was reported by Tang et al. [11] who divided the image into small sub-images (also called splats) for extracting splat features such as texture, splat area, and color. They evaluated their method based on patch and image level analysis and obtained AUC 0.96 and 0.87, respectively. For automatic detection of microaneurysm, Walter et al. [14] used morphological operations and kernel density estimation to extract a feature vector applied to a KNN, Gaussian and Bayesian risk-minimizing classifiers; their method achieved an accuracy of 88.5%.

In the past few years, deep learning approaches have been considered for this application and in 2016, Grinsven et al. [13] presented Convolutional Neural Network (CNN) architecture for detecting hemorrhage with nine layers trained by the selective misclassified negative samples. Their algorithm obtained AUC of 0.89 and 0.97 for two different datasets. In 2016, Shan and Li [15] used a patch-based analysis method to detect microaneurysm and applied a stacked sparse auto-encoder to distinguish between those two groups and they obtained 91.38% accuracy.

The success of diagnosis of DR requires the detection of all the three signs: exudate, hemorrhage and microaneurysm. While some of the studies reported earlier achieved acceptable performance for detection of single pathological sign, they were not suitable for identification of all the three signs simultaneously. Agurto et al. [26] used multiscale amplitude-modulation-frequency-modulation (AM-FM) method for extracting texture features from segmented retinal images to differentiate between groups with and without DR. To distinguish between these two groups, they computed distance metrics between the texture features. While they identified the segments with DR signs, the method did not discriminate between the three DR signs, which is essential for treatment planning. In 2017, Tan et al. [24] proposed a ten layers CNN architecture for DR sign detection. Their proposed network achieved a sensitivity of 0.87 for exudate detection, but this was only 0.62 and 0.46 for detection of hemorrhage and microaneurysm, respectively. Another limitation of this study was that they detected individual patches but did not consider the entire image which may explain the poor sensitivity due to misclassification of the background (with no pathological sign). Table 1 compares performance of the pervious methods for detection of exudate, hemorrhage and microaneurysm.

**Table 1:** Comparison between performance of the pervious methods for detection of exudate, hemorrhage and microaneurysm

Methodology	Exudate		Hemorrhage		Microaneurysm	
	sensitivity	specificity	sensitivity	specificity	sensitivity	specificity
Tan et al. [24]	0.87	0.98	0.62	0.98	0.46	0.97
Sinthanayothin et al. [29]	0.88	0.99	0.77	0.88	0.77	0.88
Grandet et al. [30]	0.94	–	0.89	–	–	–
Naqvi et al. [43]	0.92	0.81				
Walter et al. [23]	0.92	–	–	–	–	–
Fraz et al. [8]	0.92	0.81	–	–	–	–
Sopharak et al. [18]	0.82	0.99	–	–	–	–
Prentašić et al. [44]	0.78	–	–	–	–	–
Welfer et at. [17]	0.7	0.98	–	–	–	–
Niemeijer et al. [21]	–	–	0.31	–	0.31	–
Fleming et al. [20]	–	–	–	–	0.63	–
Walter et al. [14]	–	–	–	–	0.88	–
Garcia et al. [28]	–	–	0.86	–	0.86	–
Quellec et al. [19]	–	–	–	–	0.89	–
Bae et al. [16]	–	–	0.85	–	–	–
Walter et al. [14]	–	–	–	–	0.88	–

The patch-based analysis has been commonly used for CNN-based retinal image analysis [24, 33]. However, this approach can lead to disparity in the size of the sign due to patch size [24], and the inexact evaluation because of the focus on the pathological signs without considering the neighborhood and the background. While there are studies that have separated the background from the microaneurysm, and there are other studies that have accurately contoured the exudate, these perform analysis for one sign rather than all 3. Such an approach can lead to the detection with overlaps between the three signs. Another shortcoming is that while there are a number of isolated techniques that perform image enhancement, detect the presence of DR signs and perform processing to contour the signs, there is no framework that covers all the aspects.

In this study, the framework for a complete CNN-based system has been described for automatic and simultaneous detection and segmentation of exudate, hemorrhage and microaneurysm from fundus images. A ten-layered CNN architecture was designed and trained using images with annotated patches corresponding to the three signs and the background (No-sign) which was then used to obtain probability maps corresponding to each category (i.e. three sign and background). A post-processing algorithm was developed to differentiate pixels corresponding to a type of pathology from similar-looking cluttered pixels. Receiver Operating Characteristic (ROC) curve analysis was used to find a suitable threshold for differentiating between different types of pathologies This proposed framework was evaluated for both, patch and image-based analysis. Two publicly available databases were used, one was used for training while both were used for evaluation of the proposed method. The performance of the algorithm with and without probabilistic analysis was measured by taking the mean accuracy of ten repetitions.

## Materials

In this study, two public databases were used: 1- DIARETDB1, 2- e-Ophtha with total of 284 fundus images. Seventy-five images from DIARETDB1 were used for patch-based analysis, while 209 images were used for image-based analysis.

### DIARETDB1

DIARETDB1 database consists of 89 color retinal images with resolution 1500 × 1152 pixels [34]. Out of this database, 75 images were used for training the CNN while the remaining 14 images were used for testing and validating the performance of this method. In the training data, exudate, hemorrhage and microaneurysm were manually contoured by an experienced grader.

### e-Ophtha

e-Ophtha is made up of two subsets: (i) “e-Ophtha EX” which contains 47 color retina images with annotated exudate, (ii) “e-Ophtha MA” which has 148 color retina images with annotated microaneurysm [35]. In this database, there is a variation in the size and resolution of the images, ranging from 1440 × 960 to 2544 × 1696 pixels. All images were resized to the size of the DIARETDB1 (1500 × 1152 pixels).

## Methodology

**Fig. 2:**
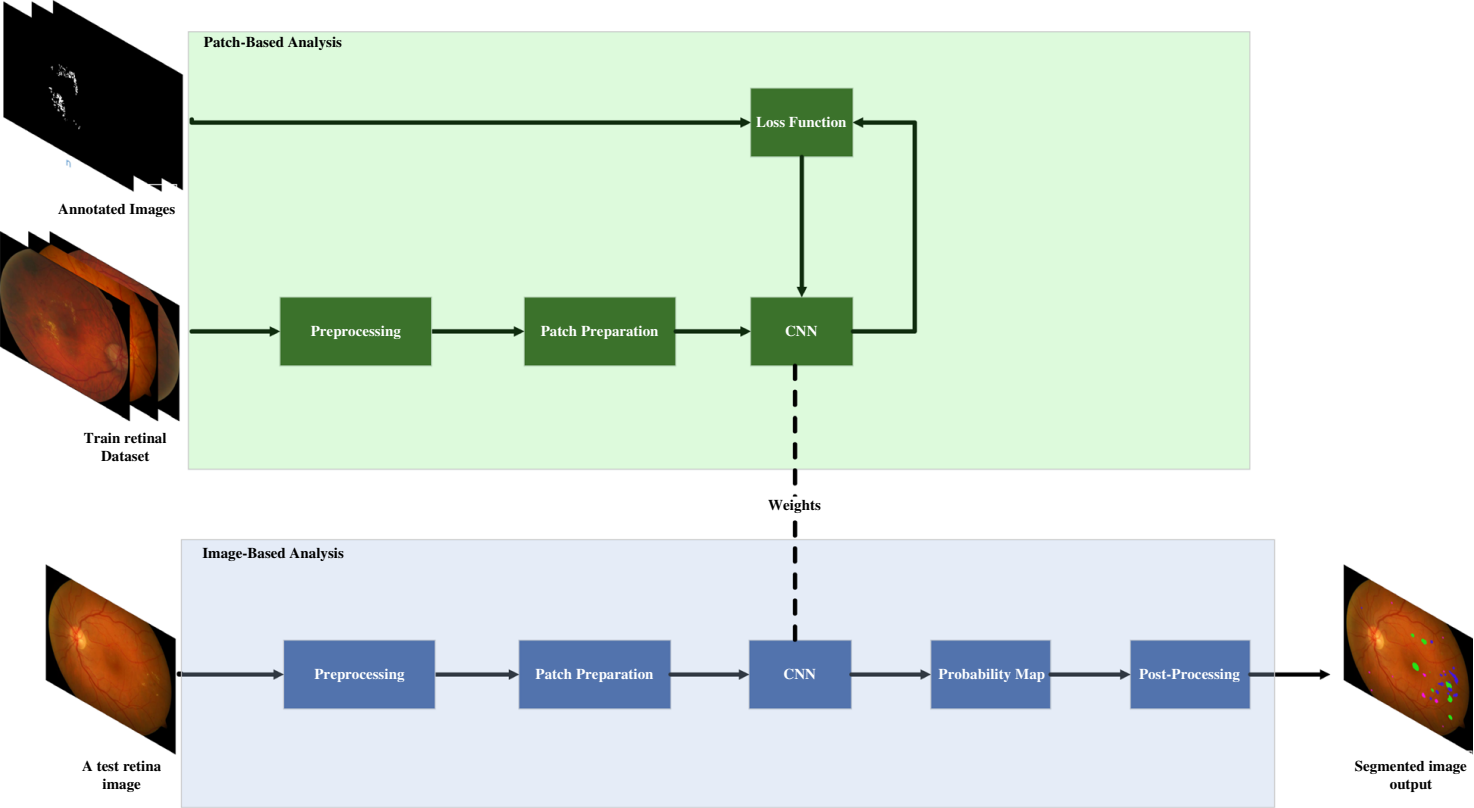
Overview of the proposed framework contains two main phases: 1) patch-based and 2) image-based analysis. The patch-based section corresponds to training and testing a CNN model to discriminate between the different DR signs. Image-based analysis of the entire image generates probability maps for each sign

The proposed framework consists of two main phases: 1) patch-based and 2) image-based analysis. The images were enhanced and then segmented in patches which were manually annotated and used to train the CNN. This trained CNN was used to analyze the other images for each pixel and a probability map was created using with which the locations of the pathological signs were identified. These images were processed to remove the isolated signs because these were noise and the spread of the signs which occurs during the earlier stages. The resultant images were compared with the manually annotated images to determine the accuracy of this method. An overview of the proposed method is shown in Fig. 2 and the steps are described below.

### Preprocessing

**Fig. 3:**
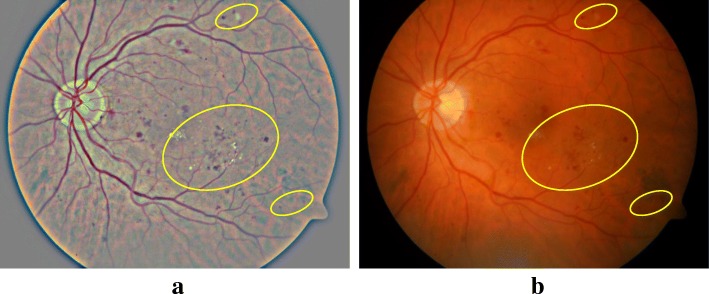
Applying the image enhancement technique on an example retina image. (**a**) Original retina image; (**b**) After image enhancement. This shows that some new lesions can be singularized by image enhancement shown by yellow annotations)

Contrast enhancement (CE) technique was used in this study to enhance the contrast between three DR pathological signs and background. In this study, the first step was to process the images using image enhancement technique [13, 36] described in eq. (1).

1 $$ {I}_{CE}=\alpha I\left(x,y\right)+\beta G\left(x,y;\sigma \right)\ast I\left(x,y\right)+\mu $$

where, *I*(*x*, *y*) is the raw image, *I*_*CE*_ the enhanced image, ∗ represents the convolution operator, *G*(*x*, *y*; *σ*) is a gaussian filter with the scale σ. The values of the *α*, *β*, *σ* and *μ* were chosen as 4, −4, 300/30 and 128, respectively based on the works by Van Grinsven [13]. This represents the subtraction of the Gaussian filtered image from the original image and highlights the contrast while *μ* gives a baseline shift of the gray scale. The result of image enhancement has been shown in Fig. 3 that revealing that some new lesions can be singularized by image enhancement, as specified by the yellow marks.

### Convolutional neural network

**Fig. 4:**
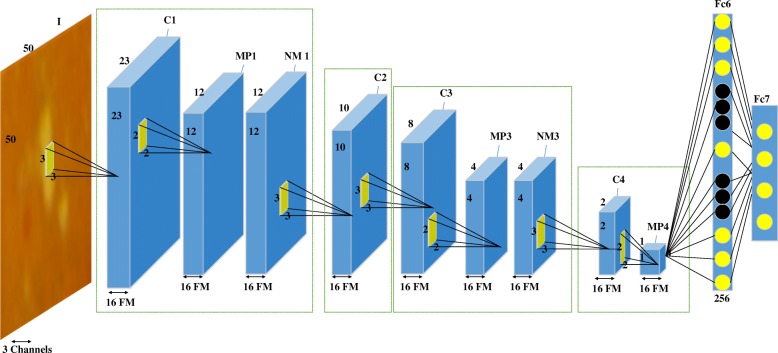
Hierarchical architecture of the proposed CNN. I: input image, C: convolutional layer, FM: feature map, MP: max pooling, NM: Normalization layer, FC: fully-connected layer

The enhanced images were segmented into patches of size of S × S which were labeled based on the ground truth images corresponding to the three pathological signs: exudate, hemorrhage, microaneurysm and background (without any pathological sign). These patches were the input to the CNN which was trained against the target labels. The choice of CNN architecture and the parameters have been described in Fig. 4.

In the proposed CNN, four convolutional layers were designed with 16 feature maps in each convolutional layer by the kernel size of 3 × 3 pixels. To avoid saturation**,** the rectified linear unit (ReLU) was employed in this study**.** The size of feature maps was reduced using Max-Pooling (MP) layer with a kernel size of 2 × 2 and the values were normalized by the normalization layers (NL) after each MP layer for faster convergence. Sixteen features were extracted from the last MP layer and fed to a fully-connected (FC) layer with 256 neurons, the output of which was given to the final stage which had four neurons corresponding to the four target classes. To avoid overfitting, drop-out algorithm with a ratio of 0.5 was used in our net design. *θ* = {*W*_*i*_, *b*_*i*_} defined as network parameters, where *w* and *b* correspond to weight and bias in the C and FC layers. For the training process, the loss function of *L*_*c*_ was defined as follows:

2 $$ {L}_c=-\frac{1}{\left|C\right|}\ \sum \limits_{i=1}^{\left|C\right|}\ln \left(p\left({D}^i|{C}^i\right)\right) $$

where |*C*| represents the number of items in the training data, *C*^*i*^ and *D*^*i*^ denote the *i*^*th*^ training sample and its label, respectively. To update *θ* parameters, stochastic gradient descent (SGD) method was used as in:

3 $$ \uptheta \left(\mathrm{p}+1\right)=\uptheta \left(\mathrm{p}\right)-\upgamma \frac{\partial {\mathrm{L}}_{\mathrm{c}}}{\mathrm{\partial \uptheta }}+\upvartheta \Delta  \uptheta \left(\mathrm{p}\right)-\mathrm{a} \upgamma \uptheta \left(\mathrm{p}\right) $$

where γ, ϑ and а denote learning rate, momentum rate and weight delay rate, respectively.

### Image analysis

**Fig. 5:**
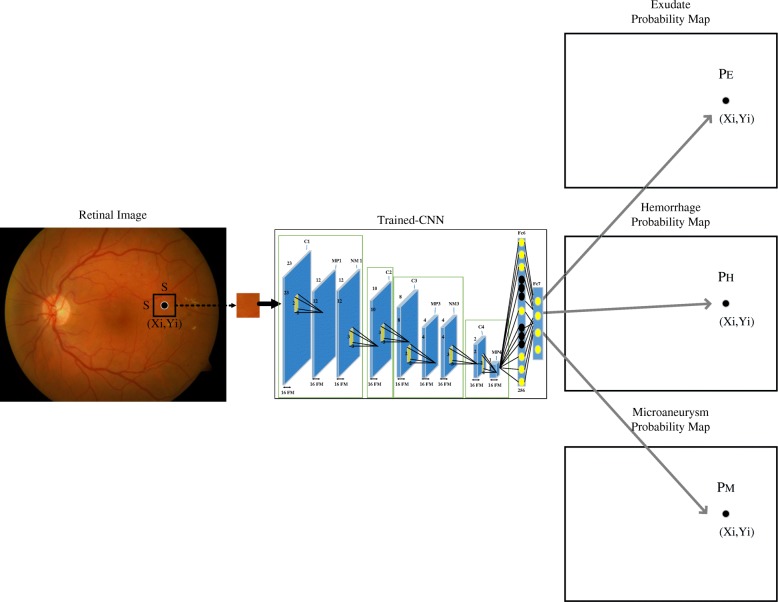
Process of generating three probability maps corresponding to exudate, hemorrhage and microaneurysm from a retina image. By taking a patch of size S × S centered around pixel (*x*_*i*_, *y*_*i*_), each patch is fed to the trained CNN that determines the membership probabilities at location (*x*_*i*_, *y*_*i*_) for the three pathological signs: i.e. exudate, hemorrhage and microaneurysm (shown by P_E,xi,yi_, P_H,xi,yi_ and P_M,xi,yi_)

In this study, pixel-based analysis of the image was performed by taking a patch of size S × S centered around pixel (*x*_*i*_, *y*_*i*_). This patch is the input to trained CNN which gives membership probabilities (range 0 to 1) at location (*x*_*i*_, *y*_*i*_) for the three pathological signs: i.e. exudate, hemorrhage and microaneurysm (shown by P_E,xi,yi_, P_H,xi,yi_ and P_M,xi,yi_). Consequently, three probability maps for the image are created and the scheme of this mapping process is shown in Fig. 5.

To identify the signs, a threshold was determined for each of the probability maps. This threshold (Th) was obtained by maximizing the receiver operating characteristics curve and used to binarize each probability map and obtain a binary map corresponding to the three signs. Overlaps were avoided by ranking the points with overlap based on the probability values. Details of this procedure are provided in section “Experiments”.

One difficulty that is faced by such methods is the appearance of redundant boundaries and cluttered pixels (False positive pixels) around the segmented signs. To overcome this shortcoming, three morphological operations: closing, opening and erosion were performed with masks of size 5 × 5, 5 × 5 and 4 × 4 pixels, respectively [37, 38]. This was followed by a rule based post-processing where signs with area of less than $\frac{S^2}{4}$ were removed.

### Validation parameters

The performance was evaluated based on false positive (FP), true positive (TP), true negative (TN) and false negative (FN) rates [39] (Table 2).

**Table 2:** Validation parameters

Parameter	Equation
Accuracy	$\frac{\mathrm{TP}+\mathrm{TN}}{\mathrm{TP}+\mathrm{TN}+\mathrm{FP}+\mathrm{FN}}$
Error rate	$\frac{\mathrm{FP}+\mathrm{FN}}{\mathrm{TP}+\mathrm{TN}+\mathrm{FP}+\mathrm{FN}}$
Positive predict value (PPV)	$\frac{\mathrm{TP}}{\mathrm{TP}+\mathrm{FP}}$
Sensitivity	$\frac{\mathrm{TP}}{\mathrm{TP}+\mathrm{FN}}$
Specificity	$\frac{\mathrm{TN}}{\mathrm{TN}+\mathrm{FP}}$

## Experiments

### Data preparation

**Fig. 6:**
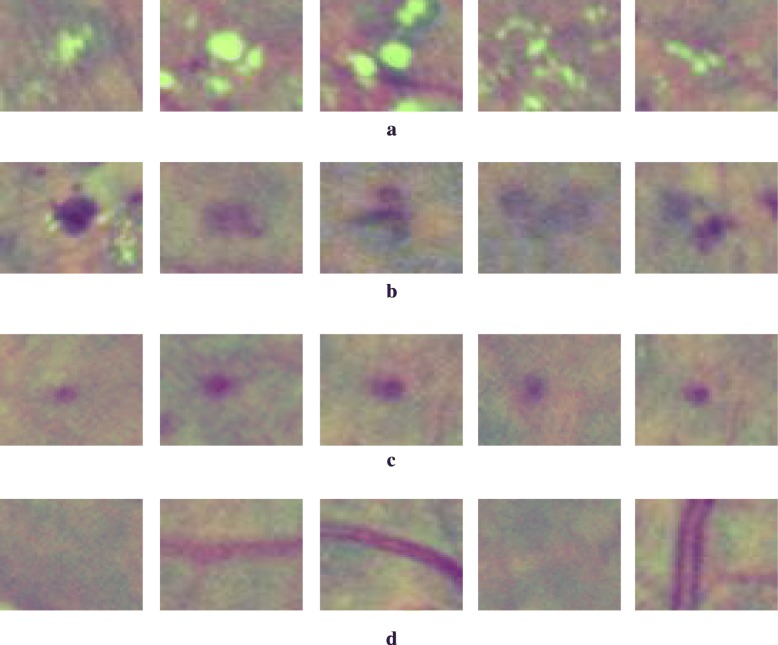
Patch examples corresponding to the four classes; (**a**) exudate. **b** hemorrhage. **c** microaneurysm. **d** no-sign

The image was segmented into patches by the size of *S* × *S*, with *S* = 50,which was determined based on the smallest pathological signs in these images. Patches corresponding to the signs were manually extracted from 75 retina images of the DIARETDB1 database and used for the training the network. These resulted in 22,719, 18,882 and 17,824 patches for exudate, hemorrhage and microaneurysm and 50,518 patches with no pathological signs (No-Sign). The No-Sign patches contained vessels, background tissue and optic nerve head. There was no overlap between each to adjacent patch. To increase the robustness of the algorithm, data augmentation was performed using both horizontal and vertical filliping and rotating [40, 41]. Figure 6 shows patch examples corresponding to four classes and Table 3 summarizes the number of patches considered for the training (75%), validation (15%) and testing (15%) CNN.

**Table 3:** Statistics information of sign patches

	Exudate	Haemorrhage	Microaneurysm	No-Sign
Training	15,646	13,339	12,477	35,013
Validation	3353	2859	2674	7503
Test	3720	2684	2333	8002
Total Number	22,719	18,882	17,484	50,518

### Network setup

For training the CNN, optimal parameters were heuristically set and shown in Table 4.

**Table 4:** CNN setup details

CNN parameters	Optimal value
Learning Rate	0.01
Momentum	0.9
Gaussian Weight Filters	0.01
Training Batch size	128
Validation and Test Batch size	32
Solver Method	SGD
Gamma	0.1
Policy of the SGD	Step-Down
Step size of SGD	33

**Fig. 7:**
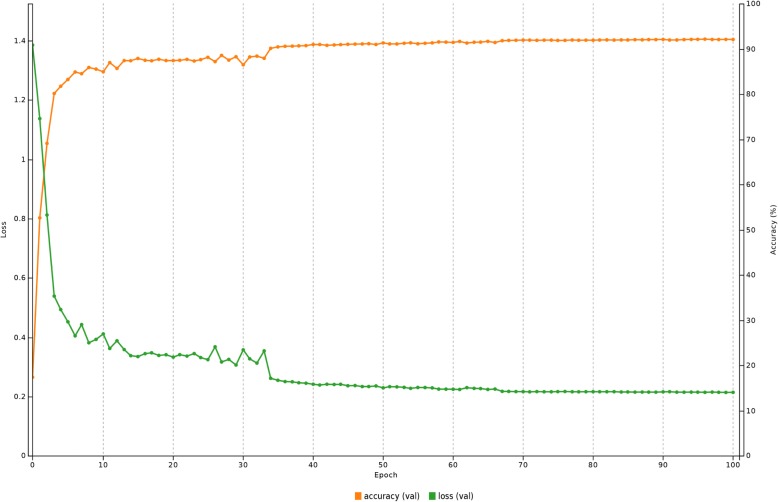
Relationship between number of training epochs with accuracy over 100 epochs. It is observed that the accuracy saturated after 43th epoch to 90% and hence was selected as the maximum number of training epochs

The maximum number of epochs was identified by repeating the training from 0 to 100 epochs and recording the accuracy and error using the validation set. It was observed that the accuracy saturated after 43th epoch to 90% and hence was selected as the maximum number of training epochs (Fig. 7). Using a GeForce GTX 1070 and Caffe platform [42] for the CNN implementation, the training process took 8 min and 23 s.

### Image analysis

**Fig. 8:**
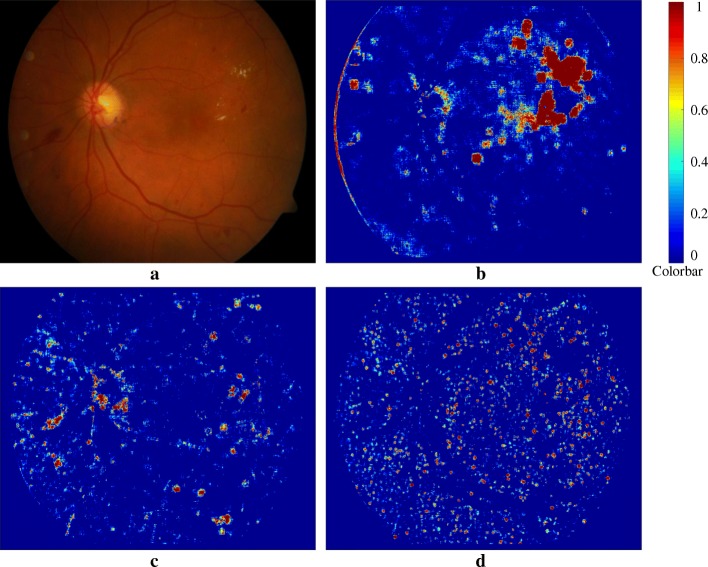
Three probability maps were generated from an example retina image: (**a**) original retina image; (**b**) Exudate probability map; (**c**) Hemorrhage probability map; (**d**) Microaneurysm probability map. Colorbar shows the severity level of a pixel belong to the sign that is ranging between 0 to 1 corresponding to blue to red color

**Fig. 9:**
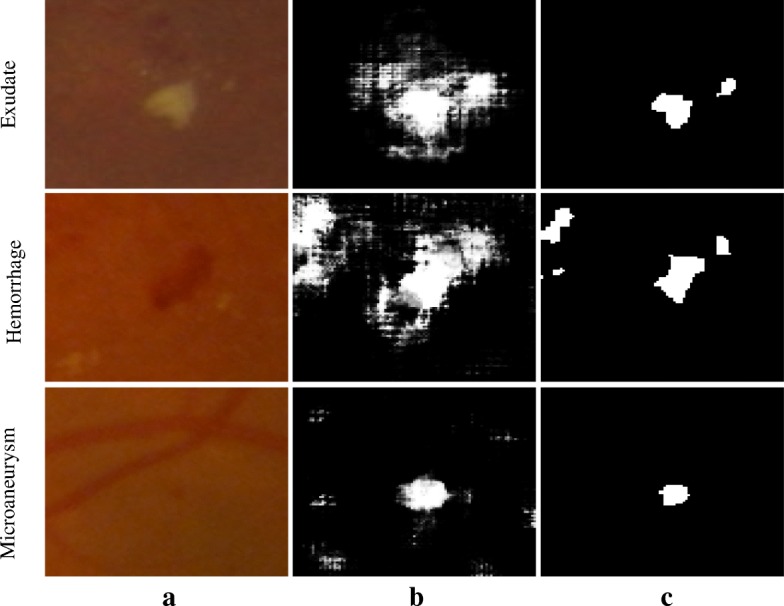
Three examples of pathological signs before and after post-processing. **a** Original image. **b** Probability map corresponding to the sign. **c** Image output after post-processing

The test image set of DIARETDB1 and all images of e-Ophtha were used to evaluate the performance of the proposed method using image-based analysis. These images were analyzed (section “Materials”) and the probability map was created of the all pixels in the image which resulted in three probability maps corresponding to exudate, hemorrhage and microaneurysm. Figure 8 shows an example with the three probability maps. Figure 9 shows the images after applying post-processing (in section “Image analysis”). It can be seen that the algorithm’s outcome accurately segmented the actual pixel’s signs from the all pixels which were assigned as potential pixels for the signs with different probability.

## Results

**Fig. 10:**
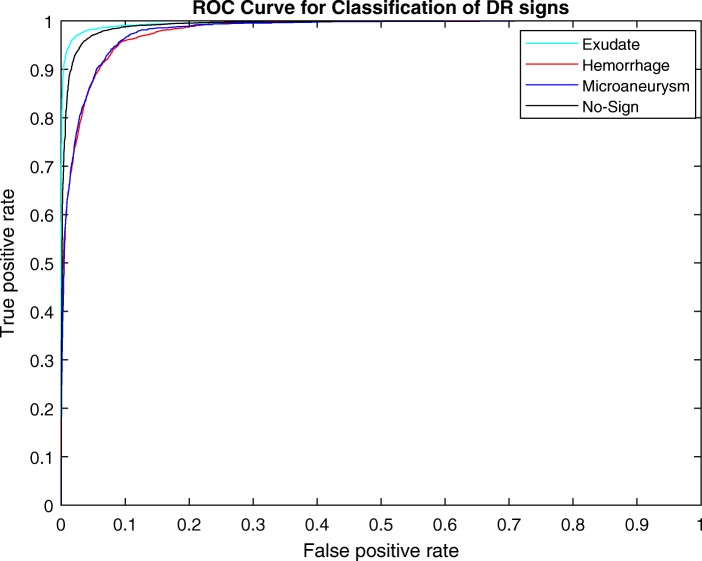
ROC curve corresponding classification of the four classes (exudate, hemorrhage, microaneurysm and no-sign)

For the patch-based evaluation, the mean results of ten repetitions for the training are described in Table 5 and Fig. 10 shows the ROC curve for the CNN performance.

**Table 5:** Sensitivity, specificity, accuracy and PPV of the proposed method in patch-level evaluation for detection of exudate, hemorrhage and microaneurysm

	Exudate	Hemorrhage	Microaneurysm	No-Sign
Accuracy	0.98	0.90	0.94	0.96
Sensitivity	0.96	0.84	0.85	0.95
Specificity	0.98	0.92	0.96	0.97
PPV	0.94	0.85	0.83	0.96

Table 5 shows the sensitivity, specificity and accuracy for the proposed method. The best results were for the exudates with sensitivity, specificity and accuracy of 0.96, 0.98 and 0.98, respectively, while that for hemorrhages was 0.84, 0.92 and 0.90, and 0.85, 0.96 and 0.94 for microaneurysm.

**Fig. 11:**
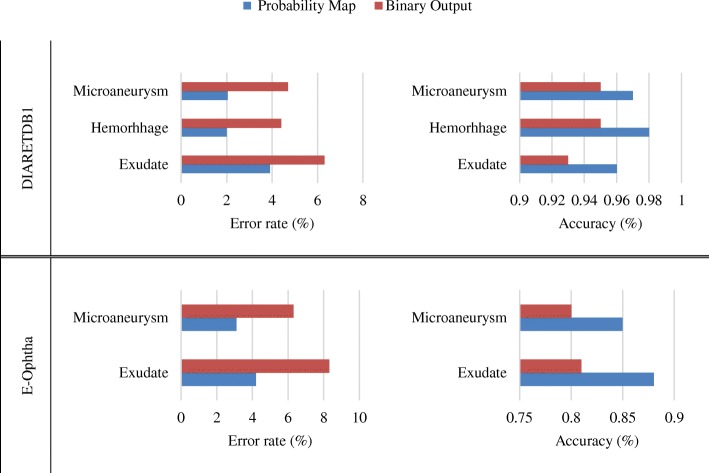
Performance of proposed framework for the sign detections using two databases (DIARETDB1 and e-Ophtha) compared to the method with binary outputs of the network

**Fig. 12:**
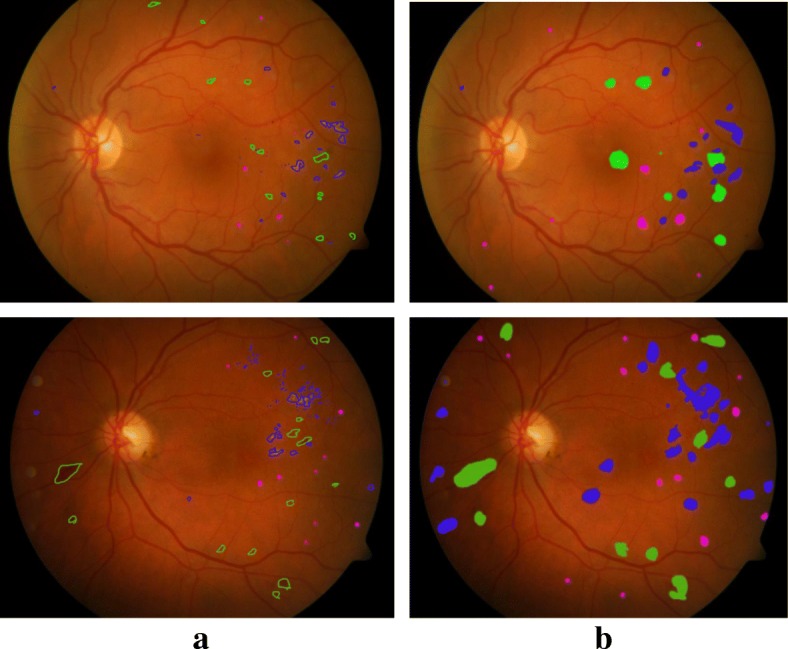
Segmentation output image of the example retina image. **a** Manually annotated images that exudate, hemorrhage, and microaneurysm signs marked by blue, green and pink color, respectively. **b** Segmented output by the proposed algorithm

For image-level evaluation, performance of the proposed method was compared to the method which used the binary outputs of the network for both datasets and shown in Fig. 11. It is observed that for DIARETDB1, the proposed method achieved the accuracy of 0.96, 0.98 and 0.97 and error rate of 3.9%, 2.1% and 2.04% for segmentation of exudate, hemorrhage and microaneurysm, respectively which shows that this technique outperforms techniques reported in literature. Similarly, there was significant improvement for exudate and microaneurysm detection in the e-Ophtha dataset with accuracy of 0.88, and 3.0 and error rate of 4.2% and 3.1%, respectively. Figure 12 shows example of a retinal image with pathological signs detected by the proposed algorithm.

## Discussion

This study has presented a CNN-based framework to analyze the retina fundus images for detection of pathologic signs indicative of DR: exudate, hemorrhage and microaneurysm. The images were first pre-processed to enhance the contrast and then segmented in patches which were then manually annotated and used for training the CNN network. This network was then used to determine the probability for each pixel to belong to the four classes of exudate, haemorrhage, microaneurysm, and background (no pathologic sign). The resultant probability map was then used to determine the locations of all the three types of pathological signs corresponding to DR. The isolated signs and the spread due to convolution were automatically removed in a post-processing step described earlier.

The results show that there was a difference in the accuracy, sensitivity and specificity when using the two databases: DIARETDB1 and e-Ophtha which could be because the CNN was trained using only DIARETDB1. Compared to previous works in which the two databases were used (Table 1), the performance of the proposed approach was higher. It also observed that average sensitivity and specificity for detecting exudates (0.96 and 0.98) is higher than for hemorrhage and microaneurysm. According to Table 1, most of the previous studies suffer from poor sensitivity, particularly for discrimination between hemorrhages and microaneurysms. Comparing our results with the work by Tan et al. [24] shows that our method achieved significantly better sensitivity for detection of hemorrhage (0.84 vs 0.62) and microaneurysm (0.85 vs 0.46), although the specificity is similar. Our method also obtained better performance for both, sensitivity and specificity, for detection of the three DR signs when compared to the work by Sinthanayothin et al. [29].

Our method simultaneously detects the three pathological signs with improved performance compared to previous studies where only one sign was considered. This makes it suitable for more reliable detection of DR because when the signs are identified individually, there is the potential error of identifying the same region for multiple signs. This method performs comprehensive analysis and detects all the three signs simultaneously. The other study that attempted the simultaneous detection of the three signs was by Tan et al. [24] which suffered from poor performance.

One innovation of this method is the use of score values obtained from the softmax layer instead of using the binary output of the network. This results in the generation of the probability map of the locations of the pathological signs on the image, which with suitable post-processing reduces the error rate in the size of the signs.

The first significant strength of this study the significant strength of the study is that we considered two different publicly available databases, with the training done on one and the testing on both with comparable results. The second strength of this study is that fundus images were analyzed using both, patch and image-based analysis, and the results show that this method is significantly better than other studies. The third strength is that this method simultaneously identifies the three different pathological signs on the images which makes it suitable for automatic detection of diabetic retinopathy because when the signs are identified individually, there is potential error when the same region is identified for multiple signs.

A limitation of this study is that it is unable to differentiate between hemorrhages and microaneurysms if there is an overlap between these. This is also a limitation of the dataset because overlaps in the original images have not been labeled. Another limitation is that the database of 284 images was imbalanced with very few images with hemorrhages. There is the need for further testing of this method for databases belonging to different demographics to determine the suitability for different societies.

## Conclusion

This paper reports a CNN based framework for the analysis of retinal images to detect the three major signs of diabetic retinopathy: exudates, hemorrhages and microaneurysms. The novelty of this system is that it uses the softmax output of the layers to generate the probability map for the three pathologic signs of DR which is then used to segment the fundus image and identify the signs. The system was trained using one dataset and tested on two datasets which shows the universality of the approach. The results show that such a system can be used for automatic analysis of fundus images for the detection of diabetic retinopathy without requiring a large dataset for training the network.

## Abbreviations

AM-FM: Multiscale amplitude-modulation-frequency-modulation
AUC: Receiver operating characteristics
CE: Contrast enhancement
CNN: Convolutional neural network
DR: Diabetic retinopathy
FC: Fully-connected
FN: False negative
FP: False positive
MP: Max-Pooling
NL: Normalization layers
ReLU: Rectified linear unit
ROC: Receiver operating characteristic
SVM: Support vector machine
TN: True negative
TP: True positive
